# DNA Repair-Based Gene Expression Signature and Distinct Molecular Subtypes for Prediction of Clinical Outcomes in Lung Adenocarcinoma

**DOI:** 10.3389/fmed.2020.615981

**Published:** 2020-11-27

**Authors:** Bin Hu, Di Liu, Yinqiang Liu, Zhixi Li

**Affiliations:** ^1^Department of Thoracic Surgery, Sichuan Cancer Hospital & Institute, Sichuan Cancer Center, The Affiliated Cancer Hospital, School of Medicine, University of Electronic Science and Technology of China, Chengdu, China; ^2^Department of Thoracic Surgery, Guizhou Provincial People's Hospital, Guiyang, China; ^3^Department of Thoracic Surgery, The First Affiliated Hospital of Kunming Medical University, Kunming, China; ^4^Lung Cancer Center, West China Hospital of Sichuan University, Chengdu, China

**Keywords:** lung adenocarcinoma, DNA repair, risk score, nomogram, molecular subtype, clinical outcomes

## Abstract

**Objective:** To conduct a robust prognostic gene expression signature and characterize molecular subtypes with distinct clinical characteristics for lung adenocarcinoma (LUAD).

**Methods:** Based on DNA repair genes from the GSEA database, a prognostic signature was conducted in the TCGA-LUAD training set via univariate and multivariate cox regression analysis. Its prediction power was validated by overall survival analysis, relative operating characteristic (ROC) curves and stratification analysis in the GSE72094 verification set. Involved pathways in the high- and low-risk groups were analyzed by GSEA. A nomogram was built based on the signature and clinical features and its performance was assessed by calibration plots. LUAD samples were clustered via the ConsensusClusterPlus package. The differences in clinical outcomes, single nucleotide polymorphism (SNP) and sensitivity to chemotherapy drugs between molecular subtypes were analyzed.

**Results:** A 13-DNA repair gene-signature was constructed for LUAD prognosis. Following validation, it can robustly and independently predict patients' clinical outcomes. The GSEA results exhibited the differences in pathways between high- and low- risk groups. A nomogram combining the signature and stage could accurately predict 1-, 3-, and 5-year survival probability. Two distinct molecular subtypes were characterized based on DNA repair genes. Patients in the Cluster 2 exhibited a worse prognosis and were more sensitive to common chemotherapy than those in the Cluster 1.

**Conclusion:**This study proposed a 13-DNA repair gene-signature as a prognostic factor for LUAD patients, which can independently predict clinical outcomes by complement of the stage. Moreover, we characterized two LUAD subtypes with distinct clinical outcomes, somatic gene mutations, and drug sensitivity in cancer based on DNA repair genes.

## Introduction

Lung cancer is one of the leading causes of cancer-related death globally (1). Non-small-cell lung cancer (NSCLC) occupies 85% of lung cancer (1). Among all cases of NSCLC, 50% are LUAD (2). Even with surgical resection at an early stage, patients with LUAD exhibit poor clinical outcomes and high recurrence risk (3). In comparison to other subtypes, LUAD has distinct molecular biological characteristics (4). Despite advances in targeted therapy, chemotherapy is still the standard treatment of LUAD. However, the incidence of chemotherapy resistance is relatively high, which can cause relapse and therapy failure, thereby ultimately lowering patients' survival time (5). High-throughput sequencing technology has accelerated the advancement of precision medicine (6). How to effectively classify the patients with same disease into different states is of importance to achieve precision medicine, depending on the genome characteristics of an individual patient. Many gene expression-related models have been conducted for prediction and stratification of LUAD patients' prognosis. Unfortunately, none of them is applied to routine clinical practice, partly due to small sample size, immoderate data fitting, as well as deficient evidence (7).

In the various activities of life, DNA damage is inevitable in organisms. The outcome of this damage depends on the degree of DNA damage and the cell's ability to repair DNA damage (8). If the damage is not repaired in time and correctly, it may lead to abnormal cell function. DNA repair is a process of correcting mismatched bases between two single strands of DNA, removing damaged bases or sugar bases on the DNA strands, and restoring the normal structure of DNA (9). DNA repair is an important link for the body to maintain the integrity and stability of the DNA structure and ensure the continuation of life and the stability of species. There are many pathways or systems to repair DNA damage in cells. Common DNA repair pathways or systems include direct repair, excision repair, recombination repair, and damage spanning repair (10). One kind of DNA damage can be repaired through multiple pathways, and one DNA repair pathway can also participate in the repair process of DNA damage at the same time. Damage to DNA bases can lead to changes in genetic code, which can produce abnormal RNA and proteins through transcription and translation, causing cell function decline, apoptosis, and even malignant transformation (11). DNA damage can lead to the activation of proto-oncogenes and the inactivation of tumor suppressor genes. The unbalanced expression of proto-oncogene and tumor suppressor gene is an important mechanism of cell malignancy (12). Studies have confirmed that DNA repair is involved in chemotherapy resistance (13), metastasis (14), and prognosis (15) of LUAD. Hence, it is of clinical significance to characterize prognostic signatures and molecular characteristics of LUAD based on these DNA repair-related gene expression profiles.

In this study, we conducted a novel prognostic gene expression signature and characterized two molecular subtypes with distinct clinical features on the basis of DNA repair-associated genes for LUAD.

## Materials and Methods

### Selection of DNA Repair-Related Genes

One hundred and fifty DNA repair-related genes (Supplementary table 1) were retrieved from the defined gene sets of “hallmark DNA repair” pathway by the Gene Set Enrichment Analysis (GSEA) database (https://www.gsea-msigdb.org/gsea/index.jsp) (16).

### LUAD Datasets

RNA-sequencing (RNA-seq) data and clinical features of 598 LUAD samples were downloaded from The Cancer Genome Atlas (TCGA) database (https://portal.gdc.cancer.gov/) on July 13, 2020. The raw data were normalized and log2 converted. Totally, 522 LUAD patients possessed complete clinical information (stage, age, gender, and overall survival time). The TCGA-LUAD dataset was applied as the training set. Somatic gene mutations for 567 LUAD samples were also obtained from TCGA portal. GSE72094 dataset containing microarray expression profile and corresponding clinical information from 398 LUAD patients was retrieved from the Gene Expression Omnibus (GEO) database (https://www.ncbi.nlm.nih.gov/geo/) (17), which was used as the validation set. Table 1 describes the clinical features of LUAD samples in the training and validation sets, respectively.

**Table 1:** The clinical features of LUAD samples in the training and validation sets.

**Characteristics**	**Training set (*n* = 522)**	**Validation set (*n* = 398)**
Age		
<65	247	107
≥65	275	291
Gender		
Female	282	222
Male	240	176
Stage		
Stage I-II	110	321
Stage III-IV	412	72
Unknown	0	5

### Construction of a Prognostic Risk Score Based on DNA Repair-Related Genes

Firstly, we screened out prognosis-related DNA repair genes with *p* < 0.05 via univariate cox regression survival analysis in the training set. Following multivariate cox regression analysis, genes independently associated with prognosis of LUAD were selected for construction of a prognostic risk score. The risk score of each sample was calculated on the basis of the regression coefficients and expression levels of selected genes, as follows: risk score = $\sum_{i=1}^{N}\left(Expi^{*}Coei\right)$ (where *N* refers to the number of selected DNA repair genes; Expi indicates the expression levels of gene i in each LUAD sample and Coei is the regression coefficient of gene i). The cutoff value was determined according to the median value of the risk scores among all samples. Then, all patients were separated into high- and low-risk groups. The expression patterns of selected genes between the two groups were visualized into a heat map via the pheatmap package in R. Kaplan-Meier survival curves were depicted for prediction of the clinical outcomes in the two groups via the survival package in R. The differences in survival were evaluated via the log-rank test. The ROC curves were built and the area under the curves (AUCs) for 1-, 3-, and 5-year overall survival (OS) were calculated utilizing the survivalROC package in R.

### Subgroup Kaplan-Meier Survival Analysis

LUAD samples in the training and validation sets were stratified into different subgroups based on age (≥65 and <65) and gender (female and male). Then, cancer samples in each subgroup were clustered into high- and low- risk groups. The differences on prognosis between the two groups were assessed via Kaplan-Meier OS analysis, followed by log-rank test.

### Univariate and Multivariate Cox Regression Analysis

The relationships between age, gender, stage, and risk score and LUAD patients' prognosis were calculated using univariate cox regression analysis. To assess which clinical factors could independently predict the clinical outcomes of LUAD patients, we presented multivariate cox regression survival analysis. Hazard ratio (HR), 95% confidence interval (CI) and *p*-value were calculated, respectively.

### GSEA

The differences in signaling pathways between the two groups were presented by adoption of the gene sets from The Molecular Signatures Database, with the cutoff values of the number of permutations = 1,000, and a false discovery rate (FDR) <0.25.

### Development of a Predictive Nomogram

The two independent prognostic factors including risk score and stage were incorporated into the nomogram model for predicting the 1-, 3-, and 5-year survival probability. The calibration plots were depicted to evaluate the relationship between actual and nomogram-predicted survival utilizing the rms package in R.

### Molecular Subtypes of LUAD Classification Based on DNA Repair Genes

On the basis of DNA repair-related genes, LUAD samples in the training and validation sets were clustered into k (2 to 9) groups using the ConsensusClusterPlus package in R (17). The optimal k value was determined to obtain a stable cluster. The PCA package in R was utilized to observe gene expression arrays in the LUAD groups. The differences on clinical outcomes between the two clusters were assessed via Kaplan-Meier survival analysis.

### Connectivity Map (CMap) Mechanism of Action (MoA) Analysis

Differential expression analysis between high- and low-risk groups was carried out via the limma package (18). Differentially expressed genes (DEGs) were determined under the criteria of |log fold change (FC)| >1 and adjusted *p*-value < 0.01. The lists of up- and down-regulated genes were uploaded into the CMap database (build 02, https://portals.broadinstitute.org/cmap/index.jsp) (19). The connectivity between the expression of these DEGs and small molecules-induced gene expression profiles was measured. Small molecules negatively associated with the indicated genes were screened out according to negative connectivity scores and *p* < 0.05.

### Chemotherapy Drug Sensitivity Analysis

The half maximal inhibitory concentration (IC50) of six chemotherapy drugs (Cisplatin, Paclitaxel, Docetaxel, Gemcitabine, Vinorelbine and Etoposide) in each LUAD sample from TCGA database was estimated via Genomics of Drug Sensitivity in Cancer (GDSC; http://www.cancerrxgene.org/) (20) utilizing the pRRophetic package in R (21). The differences in drug sensitivity of samples between two clusters were analyzed by Wilcoxon test.

### Statistical Analysis

All statistical analyses were presented via R 3.6.3 (https://www.r-project.org/).

## Results

### Development and Validation of a Prognostic Model for LUAD Patients Based on 13 DNA Repair-Related Genes

**Figure 1:**
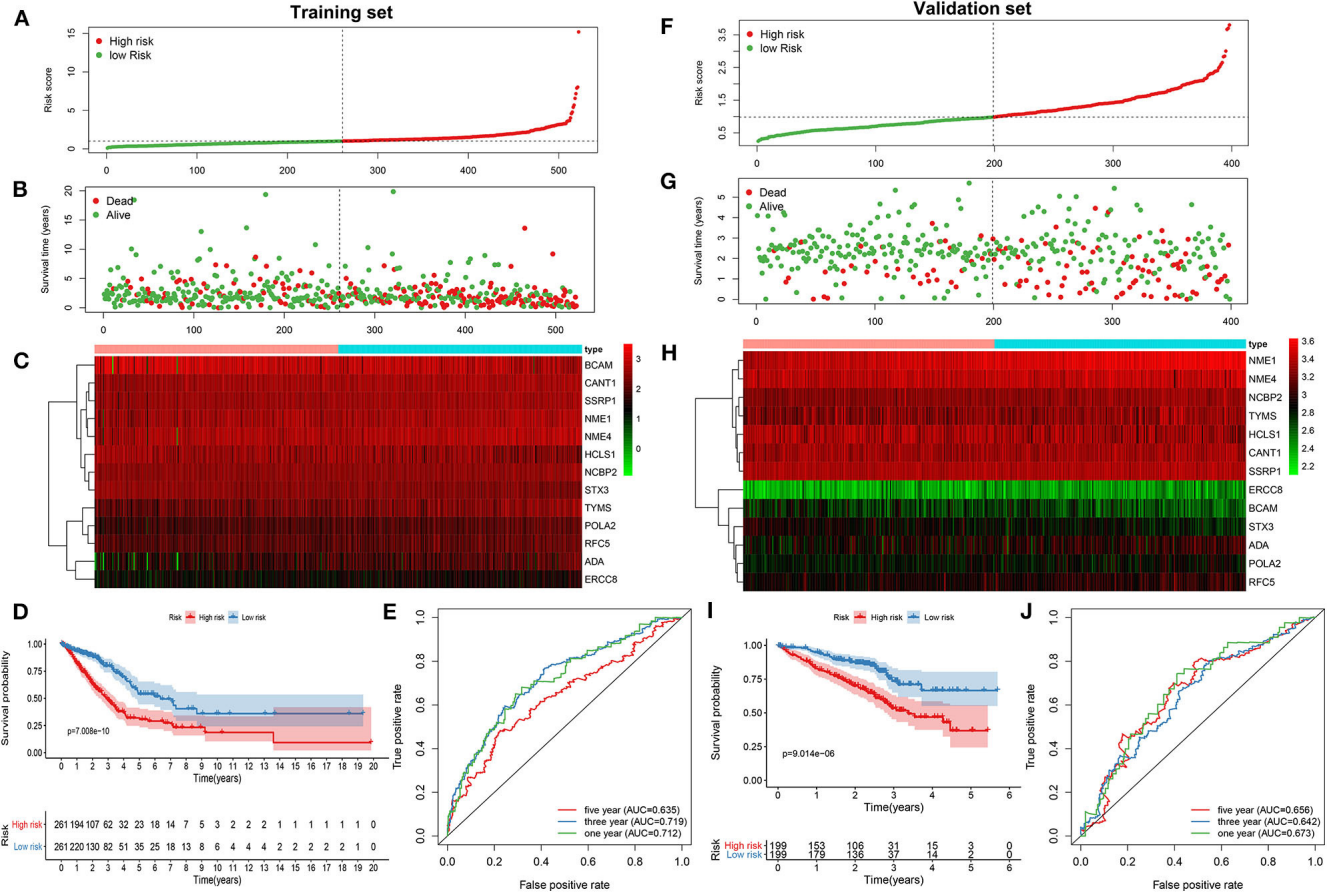
Construction and verification of a prognostic model for LUAD patients according to 13 DNA repair-related genes. In the training set, **(A)** the distribution of the risk scores among all LUAD samples. According to the median value (dotted line), LUAD samples were divided into high- (red dot) and low- risk (green dot) groups. **(B)** The distribution of survival status of all LUAD samples. Red dot is indicative of dead status and green dot indicates alive status. **(C)** Heat map depicting the expression patterns in the 13 DNA repair genes between high- and low- risk groups. **(D)** Kaplan-Meier survival curve demonstrating the OS differences between high- and low- risk groups. **(E)** The ROC curves for 1-, 3-, and 5-year OS. In the validation set, **(F)** the distribution of the risk scores among all LUAD samples. **(G)** The distribution of survival status of all patients. **(H)** Hierarchical clustering heat map depicting the expression differences in the 13 DNA repair genes between high- and low- risk groups. **(I)** Kaplan-Meier survival curve showing the OS differences between the two groups. **(J)** The ROC curves for 1-, 3-, and 5-year OS.

As tested by the univariate cox regression OS analysis, 32 DNA repair-related genes had distinct associations with OS of LUAD in the training set (all *p*-value < 0.05; Table 2). Among them, 27 genes were risk factors for LUAD prognosis [hazard ratio (HR) > 1]. Based on the multivariate cox regression OS analysis, 13 genes were independently related to prognosis of LUAD. Based on the regression coefficients and expression levels of these 13 DNA repair-related genes in each sample, the risk score was calculated as follows: 0.155162493 ^*^ expression (ADA) + (−0.172491643) ^*^ expression (BCAM) + 0.283098385 ^*^ expression (CANT1) + 0.282699006 ^*^ expression (ERCC8) + (−0.250057666) ^*^ expression (HCLS1) + 0.270360541 ^*^ expression (NCBP2) + (−0.289483144) ^*^ expression (NME1) + 0.20066966 ^*^ expression (NME4) + 0.276521771 ^*^ expression (POLA2) + (−0.491115581) ^*^ expression (RFC5) + 0.285872813 ^*^ expression (SSRP1) + (−0.582522672) ^*^ expression (STX3) + 0.300334712 ^*^ expression (TYMS). Among them, eight genes were risk factors (HR > 1) and five were protective factors (HR < 1) for LUAD prognosis. Based on the median value of the risk score, LUAD patients were separated into the high- and low- risk groups (Figure 1A). As the risk score increased, the number of patients under death status gradually increased (Figure 1B). There were distinct differences in the expression levels of the 13 genes between high- and low-risk groups (Figure 1C). LUAD patients in the high-risk group exhibited lower OS time in comparison to those in the low-risk group (*p* = 7.008e-10; Figure 1D). The sensitivity as well as specificity of the risk score was assessed via the ROC curve. The AUCs of the ROC curves for 1-, 3-, and 5- year OS were 0.712, 0.719, and 0.635, respectively (Figure 1E). We further validated the prognostic values of the risk score on the basis of 13 DNA repair-related genes in an independent GSE72094 validation set (*n* = 398). With the same calculation formular of the risk score, LUAD patients in the validation set were divided into high- and low- risk groups according to the median value of the risk scores in each sample (Figure 1F). Consistent with the training set, the number of LUAD patients with dead status was gradually augmented with the increase of the risk score (Figure 1G). The differences in the expression patterns of 13 DNA repair genes were shown in Figure 1H. As expected, LUAD patients in the high-risk group exhibited shorten OS time in comparison to those in the low-risk group (*p* = 9.014e-06; Figure 1I). The AUCs of the ROC curves for 1-, 3-, and 5- year OS were 0.673, 0.642 and 0.656, indicating the relatively high sensitivity and accuracy of the prediction model (Figure 1J).

**Table 2:** Thirty two DNA repair-related genes associated with prognosis in TCGA-LUAD cohort.

**Gene symbol**	**Hazard ratio**	**Low 95%**	**High 95%**	***P*-value**
ADA	1.276466	1.097591	1.484492	0.001531
BCAM	0.900808	0.815995	0.994436	0.038401
CANT1	1.405956	1.114076	1.774305	0.004106
CDA	1.093281	1.019235	1.172708	0.012688
DUT	1.604697	1.189018	2.165696	0.001990
ERCC1	1.377498	1.064050	1.783282	0.015047
ERCC8	1.378663	1.031678	1.842350	0.029947
FEN1	1.374169	1.151363	1.640091	0.000429
GTF2A2	1.321664	1.021947	1.709282	0.033552
HCLS1	0.824927	0.719482	0.945826	0.005813
HPRT1	1.241742	1.008485	1.528950	0.041395
LIG1	1.270208	1.018105	1.584735	0.034097
NCBP2	1.395600	1.016886	1.915356	0.039053
NME1	1.193964	1.001807	1.422979	0.047688
NME4	1.278442	1.064148	1.535891	0.008686
PNP	1.239829	1.015621	1.513533	0.034663
POLA2	1.478941	1.158915	1.887338	0.001658
POLD1	1.236396	1.003002	1.524100	0.046807
PRIM1	1.251763	1.058700	1.480034	0.008605
RAD51	1.326782	1.142959	1.540169	0.000202
REV3L	0.762314	0.599641	0.969116	0.026683
RFC2	1.247070	1.004926	1.547561	0.045008
RFC3	1.252040	1.036454	1.512468	0.019736
RFC4	1.260330	1.080214	1.470479	0.003276
RFC5	1.248384	1.001078	1.556784	0.048892
RRM2B	0.790524	0.645450	0.968205	0.023067
SAC3D1	1.239607	1.022339	1.503049	0.028913
SSRP1	1.376528	1.043073	1.816583	0.023950
STX3	0.648227	0.486480	0.863754	0.003076
TYMS	1.355027	1.169523	1.569955	5.24E-05
UMPS	1.425316	1.076630	1.886930	0.013295
ZWINT	1.246273	1.090559	1.424221	0.001225

### The Risk Score Based on 13 DNA Repair Genes Is an Independent Prognostic Factor for LUAD

**Figure 2:**
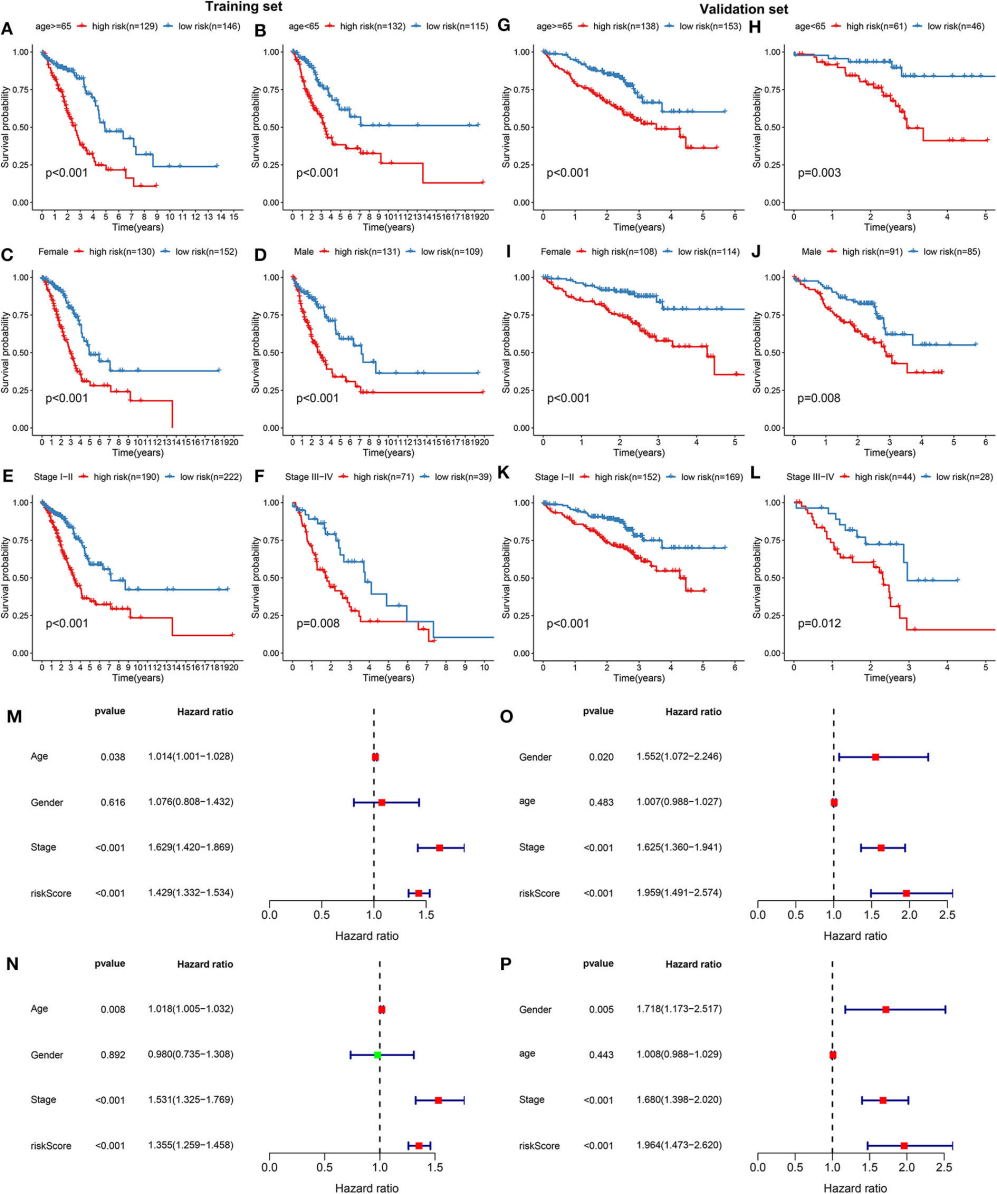
Validation the independency of the risk score based on 13 DNA repair genes for prediction of LUAD prognosis. In the training set, Kaplan-Meier curves depicted the differences on prognosis between high- and low- risk groups in different LUAD subgroups, including **(A)** ≥65, **(B)** <65, **(C)** female, **(D)** male, **(E)** stage I-II, and **(F)** stage III-IV. In the validation set, the differences on prognosis between high- and low- risk groups were confirmed in **(G)** ≥65, **(H)** <65, **(I)** female, **(J)** male, **(K)** stage I-II, and **(L)** stage III-IV subgroups. Univariate and multivariate cox regression survival analysis validated whether age, gender, stage, and risk score could independently predict the clinical outcomes of LUAD patients in the training **(M,N)** and validation sets **(O,P)**.

The prognostic characteristics of the risk score were analyzed via stratification analysis. For the training set, both in the ≥65 (Figure 2A) and <65 (Figure 2B) subgroups, patients with high risk scores exhibited a worsen prognosis in comparison to those with low risk scores (both *p* < 0.001). Regardless of whether it was a female (Figure 2C) or a male (Figure 2D) patient, high-risk score implied shorter survival time compared to low-risk score (both *p* < 0.001). Patients both at stage I-II (Figure 2E; *p* < 0.001) and III-IV (Figure 2F; *p* = 0.008) in the high-risk group had poorer prognosis compared to those in the low-risk group. Above results were confirmed in the validation set. Both ≥65 (Figure 2G; *p* < 0.001) and <65 (Figure 2H; *p* = 0.003) patients with high risk score indicated shorten OS time than those with low risk score. High risk score was indicative of shorten OS time than low risk score both for female (Figure 2I; *p* < 0.001) and male patients (Figure 2J; *p* = 0.008). Also, for patients both at stage I-II (Figure 2K; *p* <0.001) and stage III-IV (Figure 2L; *p* = 0.012), high risk score usually implied an unfavorable prognosis. We further evaluated the independence of the risk score in predicting the prognosis of LUAD patients. In the training set, the univariate cox regression analysis results demonstrated that age [*p* = 0.038 and HR (95%CI) = 1.014 (1.001–1.028)], stage [*p* = 0.038 and HR (95%CI) = 1.014 (1.001–1.028)] and risk score [*p* < 0.001 and HR (95%CI) = 1.429 (1.332–1.534)] were distinctly associated with LUAD patients' prognosis (Figure 2M). According to the multivariate cox regression analysis results, stage [*p* < 0.001 and HR (95%CI) = 1.531 (1.325–1.769)] and risk score [*p* < 0.001 and HR (95%CI) = 1.355 (1.259–1.458)] were both independent prognostic factors for LUAD (Figure 2N). The independency of the risk score for prediction of LUAD prognosis was confirmed in the validation set (Figures 2O,P). Collectively, the risk score was an independent prognostic factor for LUAD.

### Differences in Signaling Pathways Between High- and Low- Risk Groups

**Figure 3:**
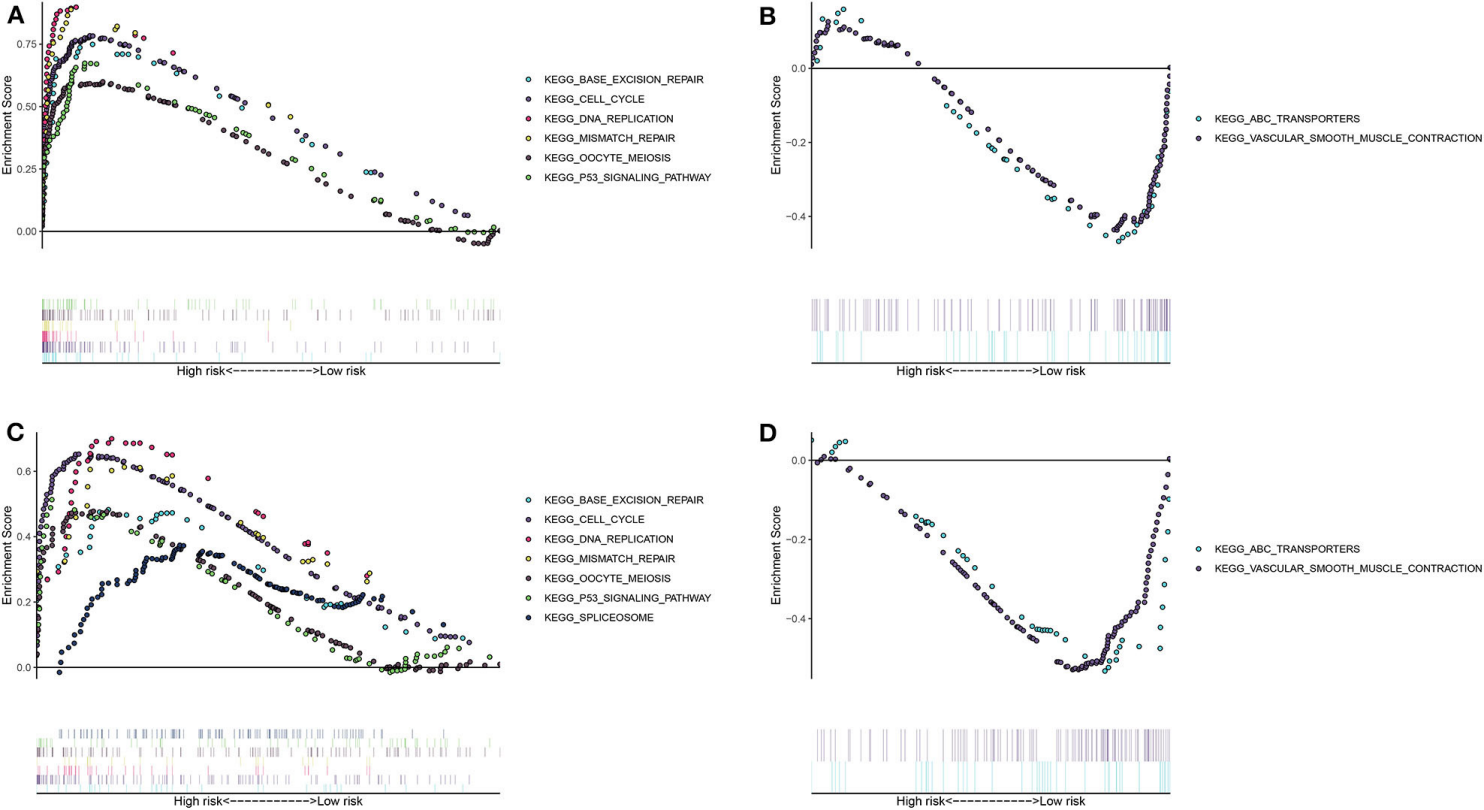
Unveiling the differences in involved signaling pathways between high- and low- risk groups. **(A,B)** Involved signaling pathways in the high- and low-risk groups in the training set. **(C,D)** Involved signaling pathways in the high- and low-risk groups in the validation set.

The differences in signaling pathways between high- and low- risk groups were analyzed via GSEA. For the training set, base excision repair, cell cycle, DNA replication, mismatch repair, oocyte meiosis, P53 signaling pathway and spliceosome were distinctly enriched in the high-risk group (Figure 3A). At the same time, ABC transporters and vascular smooth muscle contraction were significantly enriched in the low-risk group (Figure 3B). The similar enrichment results for high- (Figure 3C) and low- risk groups (Figure 3D) were confirmed in the validation set.

### Development and Verification of a Personalized Prognostic Prediction Nomogram for LUAD

**Figure 4:**
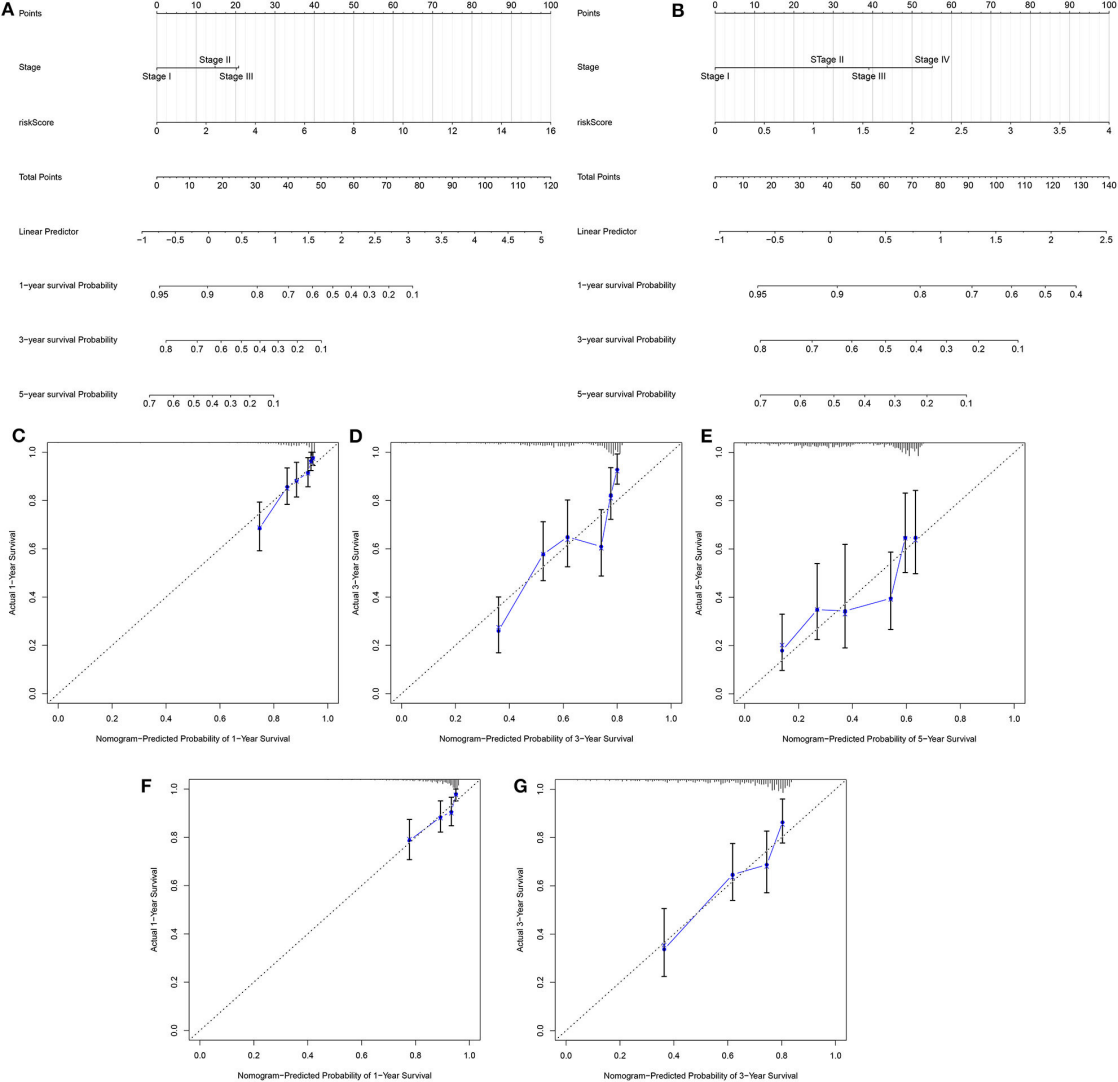
Development and verification of a nomogram for prediction of LUAD patients' 1-, 3-, and 5-year OS time. The nomogram including stage and risk score for prediction of 1-, 3-, and 5-year survival probability was conducted and verified in the **(A)** training set as well as **(B)** validation set. **(C–E)** Calibration plots showed the association between actual and the nomogram-predicted probability of 1-, 3-, and 5-year survival in the training set. **(F,G)** The association between actual and the nomogram-predicted probability of 1- and 3-year survival was confirmed in the validation set.

Two independent prognostic factors including stage and risk score were utilized for constructing the nomogram for prediction of the 1-, 3-, and 5-year survival probability in the training set (Figure 4A). Its feasibility in clinical practice was confirmed in the validation set (Figure 4B). As shown in the calibration plots, the nomogram could stably predict 1- (Figure 4C), 3- (Figure 4D), and 5-year (Figure 4E) OS in the training set. By confirmed in the validation set, 1- (Figure 4F), and 3-year (Figure 4G) OS was robustly predicted by the nomogram for LUAD patients. Taken together, the nomogram could possess the high clinical applicability for prediction of the survival probability of LUAD patients.

### Characterization of Two LUAD Molecular Subtypes With Distinct Clinical Outcomes Based on DNA Repair-Associated Genes

**Figure 5:**
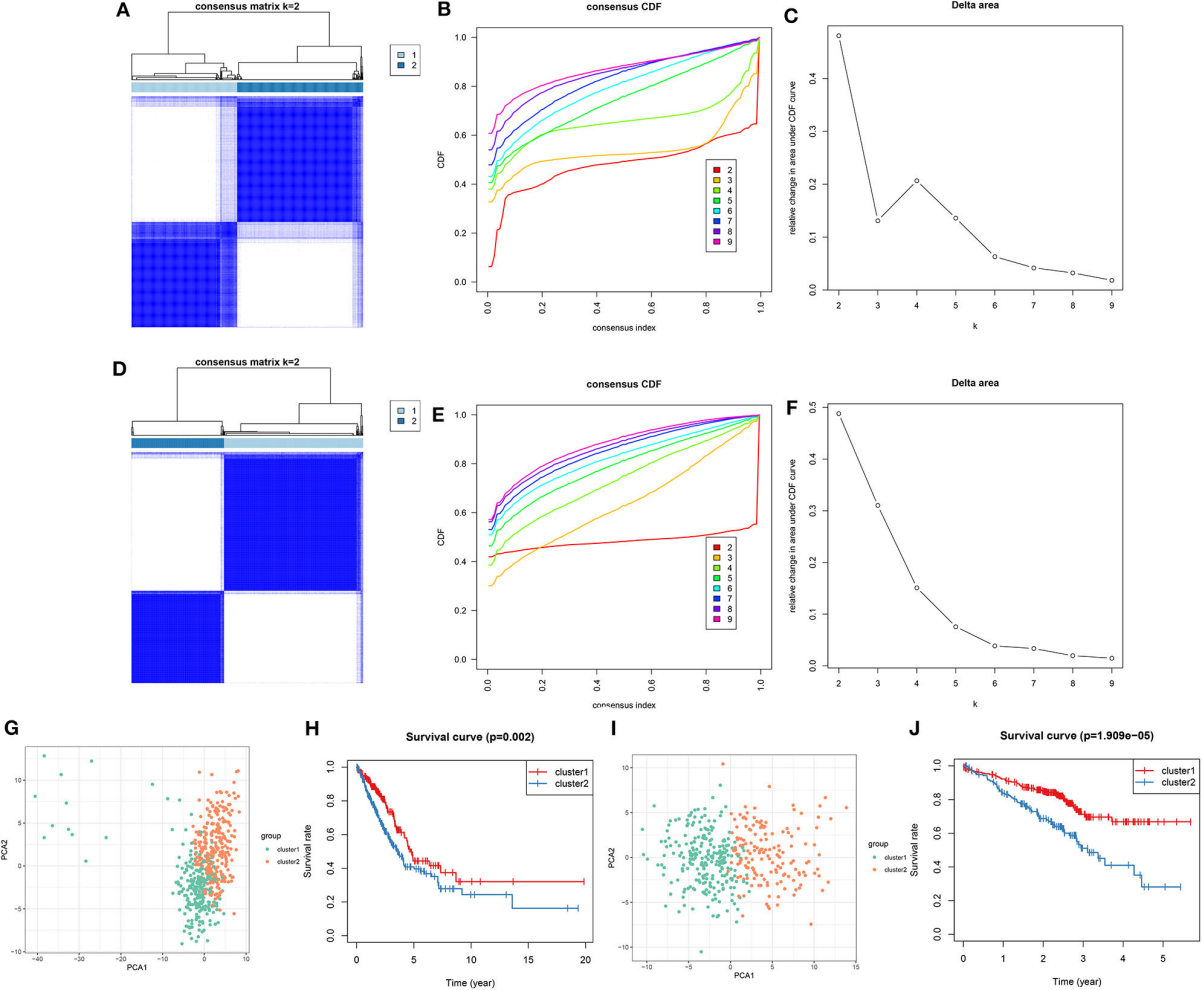
Consensus clustering of DNA repair-associated genes clustered LUAD samples into two clusters with distinct clinical outcomes. In the training set, **(A)** the heat map showing the consensus matrix when *k* = 2. **(B)** Consensus clustering cumulative distribution function (CDF) under *k* = 2–9. **(C)** Relative change in area under CDF curve. In the validation set, **(D)** the heat map depicting the correlation between cluster 1 and 2. **(E)** Consensus clustering CDF under *k* = 2–9. **(F)** Relative change in area under CDF curve. **(G)** PCA of the expression profile of DNA repair genes from cluster 1 and 2 in the training dataset. **(H)** Kaplan-Meier OS curve between cluster 1 and 2 in the training set. **(I)** PCA of the expression profile of DNA repair genes from cluster 1 and 2 in the validation dataset. **(J)** Kaplan-Meier OS curve between cluster 1 and 2 in the validation dataset.

Utilizing the ConsensusClusterPlus package, LUAD samples were clustered into different groups. When *k* = 2, two molecular subtypes were stably classified both in the training (Figures 5A–C) and validation sets (Figures 5D–F). PCA results demonstrated that there was a distinct difference in the expression profiles of DNA repair genes between the two molecular subtypes in the training set (Figure 5G). As shown in Kaplan-Meier OS curve, LUAD patients in the cluster 2 exhibited shorten OS time than those in the cluster 1 (*p* = 0.002; Figure 5H). Consistent with the training set, LUAD samples in the validation set were stably divided into two groups based on the expression profiles of DNA repair genes (Figure 5I). Moreover, patients in the cluster 2 had distinctly poorer prognosis in comparison to those in the cluster 1 (*p* = 1.909e-05; Figure 5J). Taken together, we characterized two LUAD molecular subtypes with distinct clinical outcomes according to DNA repair-associated genes.

### MoA Analysis via CMap Database

**Figure 6:**
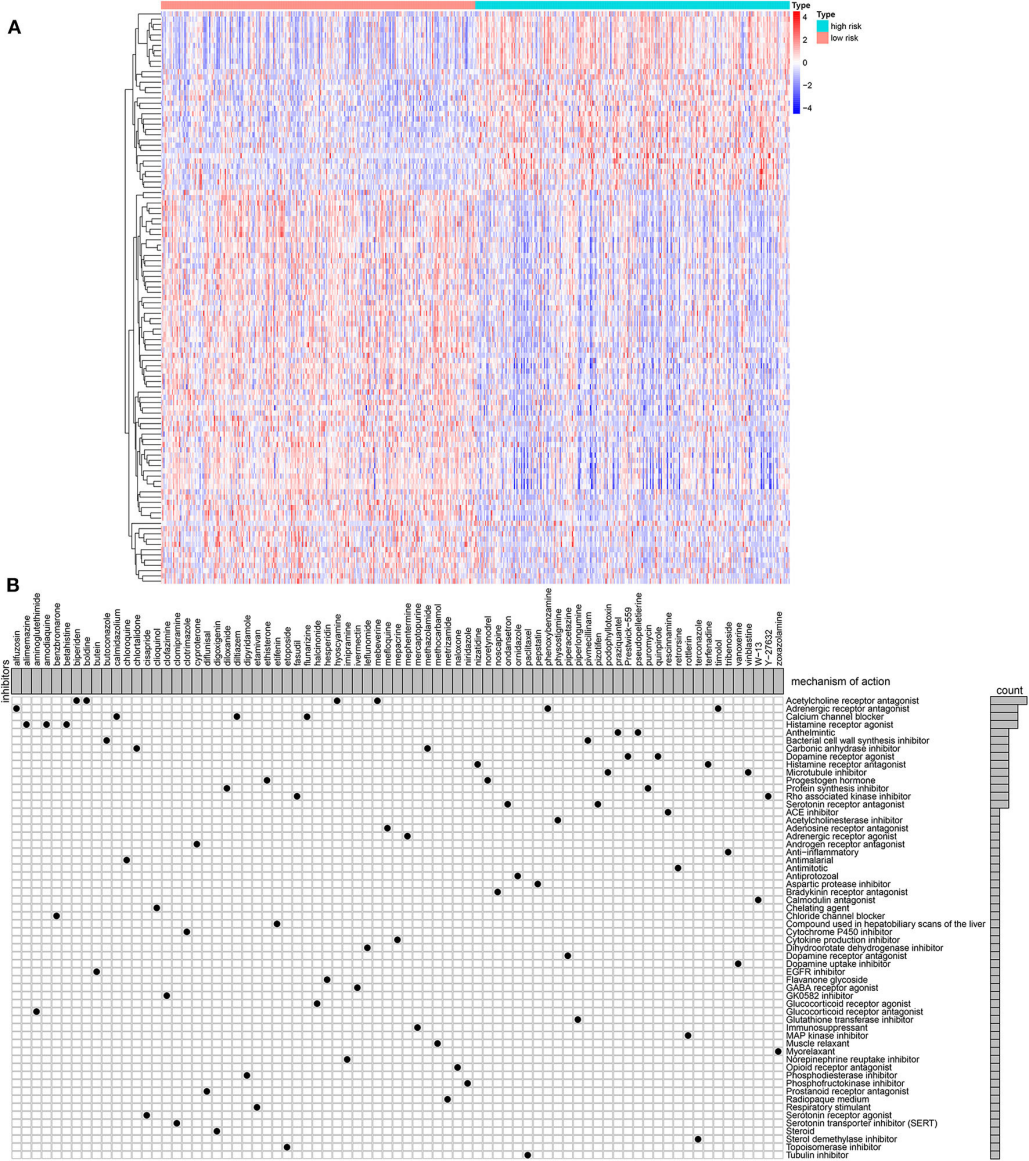
MoA analysis results via CMap database. **(A)** Heat map showing all DEGs between high- and low-risk groups. **(B)** Shared mechanism diagram of each compound in CMap.

One hundred and nine DEGs were identified between high- and low-risk groups, with the threshold of |logFC| > 1 and adjusted *p*-value < 0.01, as shown in Figure 6A and Supplementary Table 2. The up- and down-regulated genes were imported into the CMap database. The results showed that 78 small molecules were predicted to target these DEGs. Moreover, 58 kinds of mode of drug actions were distinctly enriched by MoA analysis results (Figure 6B).

### Differences in Somatic Mutations Between Two LUAD Clusters

**Figure 7:**
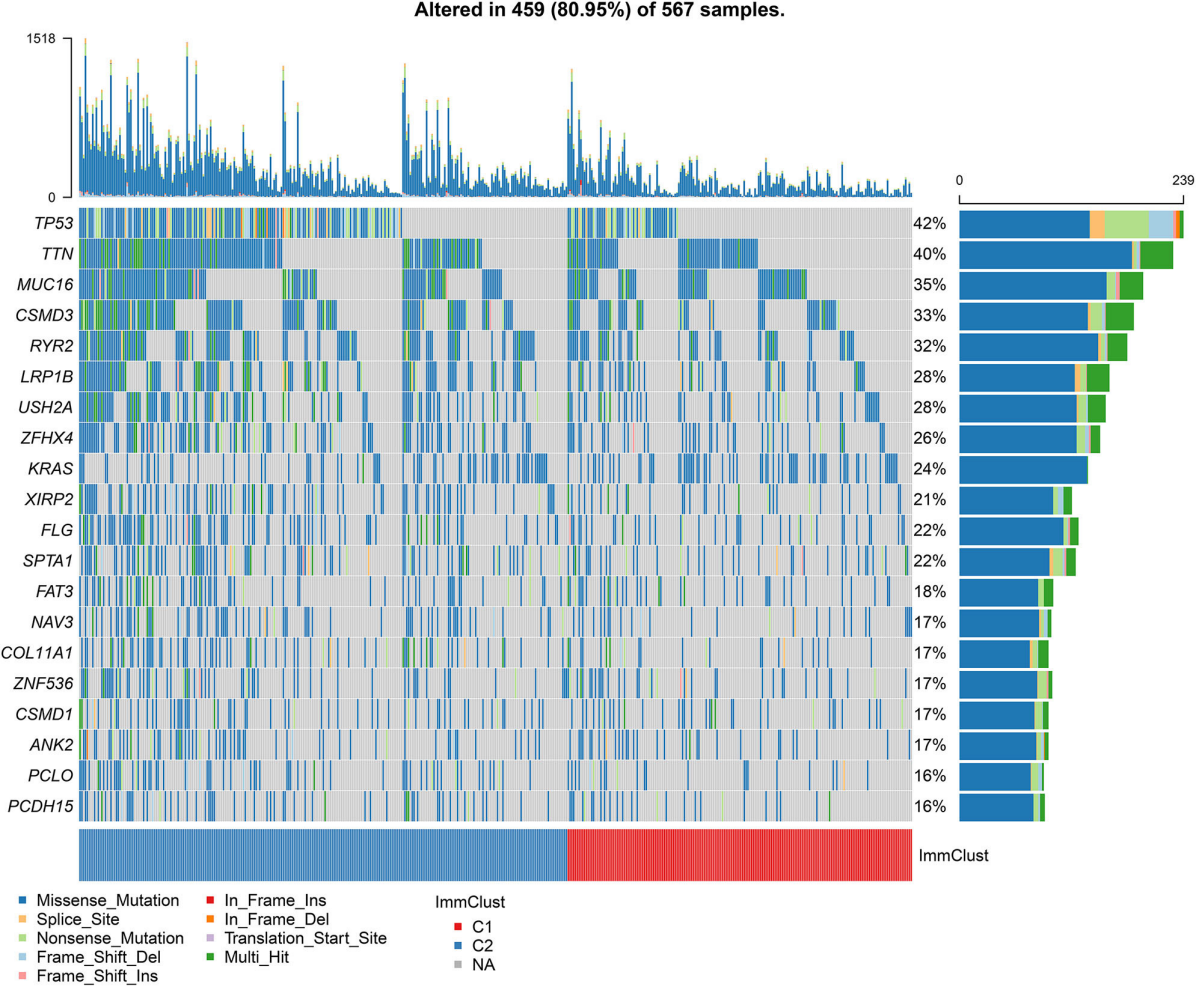
Differences in somatic mutations between two LUAD clusters from TCGA database. The 20 genes are ordered according to the frequencies of mutation. The bottom of the panel exhibits the mutation types as well as sample clusters. The right of the panel displays the mutation frequencies of the genes.

Among 567 LUAD samples from TCGA database, 459 (80.95%) occurred somatic mutations. Twenty frequently mutated genes were defined. Among them, TP53 (42%), TTN (40%), MUC16 (35%), CSMD3 (33%) and RYR2 (32%) were the five most frequently mutated genes (Figure 7). The most frequently mutation type was missense. Furthermore, the samples in the cluster 2 exhibited higher mutation levels in comparison to those in the cluster 1.

### Evaluation of the Sensitivity of Chemotherapy Drugs to Two LUAD Clusters

**Figure 8:**
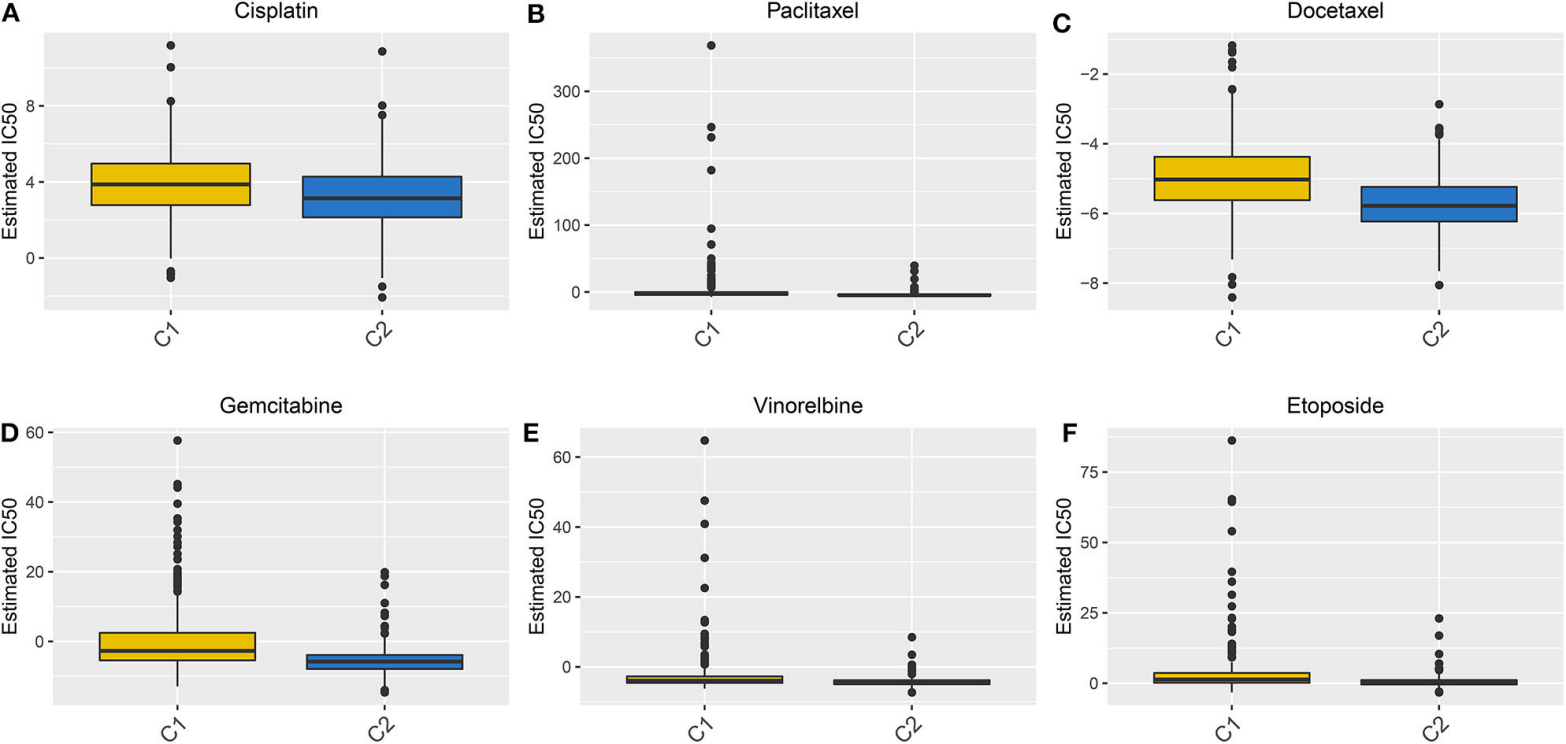
Box plots showing the differences in estimated IC50 values of six chemotherapy drugs between the two LUAD clusters. **(A)** Cisplatin. **(B)** Paclitaxel. **(C)** Docetaxel. **(D)** Gemcitabine. **(E)** Vinorelbine. **(F)** Etoposide. Each dot is indicative of the estimated IC50 value in each LUAD sample. The higher the sensitivity to the drug, the lower the IC50 value.

We evaluated the differences in the sensitivity of chemotherapy drugs between the Two LUAD clusters using the GDSC database. The estimated IC50 values of Cisplatin (Figure 8A), Paclitaxel (Figure 8B), Docetaxel (Figure 8C), Gemcitabine (Figure 8D), Vinorelbine (Figure 8E) and Etoposide (Figure 8F) were all significantly higher in samples in the cluster 1 in comparison to those in the cluster 2 (all *p* < 0.05). These findings suggested that LUAD patients in the cluster 2 could show sensitivity to these six chemotherapy drugs.

## Discussion

In this study, we constructed a robust 13-DNA repair gene-signature for LUAD patients' prognosis, which could assist the stage system to predict the clinical outcomes, thereby providing more suitable treatments. Furthermore, two molecular subtypes with distinct clinical features were built on the basis of the expression profiles of DNA repair genes, which could be applied to tailor therapeutic strategies for LUAD patients.

It has been confirmed that DNA repair is widely involved in chemosensitivity, prognosis and metastasis of LUAD (22). A thorough understanding of the expression profile of DNA repair-associated genes in LUAD specimens may provide new ideas for improvement of patients' clinical outcomes. Totally, 150 DNA repair genes were obtained from the GSEA database. Via the univariate and multivariate cox regression analysis, we constructed a 13-gene signature. Patients with high risk score were indicative of poorer OS time than those with low-risk score. The AUCs of the ROCs for 1-, 3-, and 5-year OS time confirmed its well-predictive performance, which were confirmed in the validation set. Furthermore, its predictive accuracy and independency were verified by stratification analysis and multivariate cox regression analysis. As previous research, the prognostic potential of DNA repair genes has been found in gastric cancer (23). A 7-DNA repair gene-signature can predict hepatocellular carcinoma patients' prognosis (24). We further probed into involved signaling pathways for high- and low-risk groups. As a result, different pathways were enriched in the high- and low-risk groups. Several LUAD-related pathways including base excision repair (25), cell cycle (26), DNA replication (27), mismatch repair (28), oocyte meiosis (29), P53 signaling pathway (30) and spliceosome (31) were distinctly enriched in the high-risk group. Moreover, ABC transporters (32) and vascular smooth muscle contraction (33) were significantly enriched in the low-risk group, suggesting that DNA repair genes for high- and low-risk groups participated in distinct pathways. Precision medicine largely depends on the identification of individual genomic characteristics of different LUAD patients. By combining the signature and stage, we established a nomogram for prediction of 1-, 3-, and 5-year OS. The model can accurately classify patients' prognostic risk. Based on DEGs between high- and low-risk groups, we screened 77 small molecule drugs and 58 drug mechanisms for LUAD, which should be validated by in-depth analysis.

The prediction of the therapy response is one of the main goals of precision medicine, depending largely on an unknown subset of biological characteristics. Characterization of the molecular characteristics for a specific patient is essential to alleviate heterogeneity and tailor treatment (34). By analyzing the LUAD samples from the TCGA-LUAD training set and an independent GSE72094 verification set, we characterized two LUAD molecular subtypes based on DNA repair-related gene expression profiles. Kaplan-Meier OS analysis results demonstrated that LUAD patients in the cluster 2 exhibited worsen clinical outcomes than those in the cluster 1. Chemotherapy is the first choice for LUAD patients in the advanced stage, but its response rate is very low (35). Chemoresistance contributes to the short survival time of LUAD patients following initial chemotherapy (32). It has been estimated that chemotherapy can only reduce the deaths of lung cancer patients by 4% following 5 years in comparison to the untreated group (36). Hence, it is of importance to identify a specific molecular subtype of LUAD that could be sensitive to chemotherapy. In this study, patients in the cluster 2 may be more sensitive to six chemotherapy drugs (Cisplatin, Paclitaxel, Docetaxel, Gemcitabine, Vinorelbine, and Etoposide) compared to those in the cluster 1, which should be validated in future clinical trials.

However, there are several limitations in our study. Firstly, the signature and molecular subtypes were constructed by a retrospective study. In future studies, their predictive power will be verified in large-scale prospective research. Secondly, due to lack of SNP data in other databases, the differences in SNP between the two molecular subtypes were verified in independent datasets. Taken together, the signature and molecular subtypes that we constructed could be used to improve the current risk stratification of LUAD.

## Conclusion

Collectively, this study constructed a 13-DNA repair gene-signature for LUAD prognosis. Following validation, this signature can accurately and independently predict patients' clinical outcomes. A nomogram combining the signature and stage was established as an individual clinical prediction tool. According to DNA repair gene expression profiles, two molecular subtypes were characterized, with distinct clinical outcomes, somatic gene mutations as well as sensitivity to chemotherapy drugs, which may be used to guide clinical treatment decisions.

## Data Availability Statement

The original contributions presented in the study are included in the article/Supplementary Materials, further inquiries can be directed to the corresponding author.

## Author Contributions

ZL conceived and designed the study. YL and BH conducted most of the experiments and data analysis, and wrote the manuscript. DL participated in collecting data and helped to draft the manuscript. All authors reviewed and approved the manuscript. All authors contributed to the article and approved the submitted version.

## Conflict of Interest

The authors declare that the research was conducted in the absence of any commercial or financial relationships that could be construed as a potential conflict of interest.

## Supplementary Material

Supplementary Table 1 A total of 150 DNA repair-related genes.

Supplementary Table 2 109 DEGs between high- and low-risk groups with the threshold of |logFC| >1 and adjusted *p*-value < 0.01.

## Abbreviations

LUAD: lung adenocarcinoma
ROC: relative operating characteristic
SNP: single nucleotide polymorphism
NSCLC: non-small-cell lung cancer
LUAD: lung adenocarcinoma
GSEA: Gene Set Enrichment Analysis
RNA-seq: RNA-sequencing
TCGA: The Cancer Genome Atlas
GEO: Gene Expression Omnibus
AUCs: area under the curves
OS: overall survival
HR: hazard ratio
CI: confidence interval
FDR: false discovery rate
CMap: Connectivity Map
MoA: mechanism of action
DEGs: differentially expressed genes
FC: fold change
IC50: half maximal inhibitory concentration
GDSC: Genomics of Drug Sensitivity in Cancer.
